# Comparative effect of olive oil and flaxseed oil on drug induced hepatotoxicity in rats

**DOI:** 10.1002/fsn3.4423

**Published:** 2024-10-23

**Authors:** Waqas Ahmad Khan, Muhammad Inam‐ur‐Raheem, Hina Rasheed, Muhammad Abdullah Butt, Farhan Saeed, Muhammad Afzaal, Faiyaz Ahmed, Noor Akram, Aasma Asghar, Gebremichael Gebremedhin Hailu

**Affiliations:** ^1^ National Institute of Food Science and Technology (NIFSAT), University of Agriculture Faisalabad Pakistan; ^2^ Department of Food Science Government College University Faisalabad Faisalabad Pakistan; ^3^ Department of Basic Health Sciences, College of Applied Medical Sciences Qassim University Buraydah Saudi Arabia; ^4^ Food Safety & Biotechnology lab, Department of Food Science Government College University Faisalabad Faisalabad Pakistan; ^5^ Department of Nutritional Sciences Government College University Faisalabad Faisalabad Pakistan; ^6^ Food Technology and Process Engineering Oda Bultum University Chiro Ethiopia

**Keywords:** anti‐inflammatory, antioxidant, chemical toxicity, detoxifying chemicals, glucose metabolism, hepatotoxicity, lipid profiles, liver diseases

## Abstract

This study investigates the hepatoprotective effects of olive oil and flaxseed oil on chemically induced hepatotoxicity in rats by evaluating key biochemical parameters, including free fatty acids, iodine value, liver enzymes (ALP, ALT, AST), cholesterol, triglycerides, and HDL levels. The results demonstrated significant improvements in these markers with the administration of olive oil and flaxseed oil, either individually or in combination. The free fatty acid percentages and iodine values were consistent with the known nutritional properties of the oils. Notably, the combination of olive oil and flaxseed oil yielded the most substantial benefits, reducing ALP (115.53 ± 0.44 U/L), ALT (50.77 ± 1.46 U/L), and AST (52.12 ± 0.36 U/L) levels while improving lipid profiles by lowering cholesterol (135.4 ± 1.43 mg/dL) and triglyceride levels (57.14 ± 2.35 mg/dL) and increasing HDL levels (49.20 ± 0.45 mg/dL). These findings suggest that olive oil and flaxseed oil, due to their antioxidant and anti‐inflammatory properties, can effectively mitigate liver damage and promote lipid metabolism. The study supports the potential therapeutic application of these oils in managing liver health and preventing hepatotoxicity.

## Abstract 2

In this study, flaxseed oil and olive oil were analyzed and tested for their therapeutic effects over a 28 day period using rats. Hepatotoxicity was induced (by CCL4) in five groups, and the oils were administered accordingly. The results showed that both oils significantly reduced elevated liver enzymes (ALT, AST, and ALP) and improved lipid profiles, except for HDL.

## 1 INTRODUCTION

The liver is a crucial organ responsible for multiple functions, including detoxification and vitamin storage. Liver issues can lead to serious health problems and are often caused by exposure to toxic chemicals, unsanitary water, poor dietary habits, unhygienic environments, intake of toxic substances, and excessive use of painkillers and alcohol (Ekebas et al., 2019). In Pakistan, Hepatitis B and Hepatitis C are prevalent, with one in ten individuals suffering from liver disease. The prevalence rate of Hepatitis C is 4.8%, while Hepatitis B is 2.5%. Non‐alcoholic fatty liver disease (NAFLD) is also a significant concern. According to the World Health Ranking, Pakistan ranks 60th in liver injury patients and liver disease is the 11th leading cause of death in the country (WHR, 2018). Liver damage can also result from reactive oxygen or nitrogen species (Clemens et al., 2019).

Liver diseases are a significant issue in Pakistan today. However, Prophet Muhammad said, “There is no disease that God has created, except that He also has created its treatment” (Weber, 2023). To reduce elevated liver enzymes, antioxidant and anti‐inflammatory agents like olive oil and flaxseed oil can be beneficial due to their nutritional properties, which help mitigate hepatotoxicity.

Olive oil, derived from the *Olea europaea L*. plant, is rich in phenols and phytochemicals and is known for its therapeutic properties against diseases like colorectal, renal, and prostate cancer. It contains squalene, which is beneficial for human health (Abenavoli et al., 2018). Olive oil is a major source of bio‐phenolic compounds and fats used in pharmaceuticals and nutraceuticals. It has anti‐inflammatory, antigenotoxic, anticancer, and antimutagenic properties and is rich in antioxidants such as carotenes and tocopherols, which preserve its quality and contribute to health (Tufarelli et al., 2017). Its chemical composition varies with climate, geography, cultivation type, and extraction process (Lazzerini et al., 2017). Nutritionally, olive oil contains monounsaturated fatty acids, vitamin K, vitamin E, and other fatty acids like linolenic acid and palmitic acid (Guo et al., 2018).

Flaxseed oil, from the *Linum usitatissimum* plant, is known for its antioxidant properties and is widely used as a cardioprotective therapeutic food (Guimarães et al., 2013). It has hypocholesterolemic effects, increasing HDL levels and decreasing LDL levels (Ahlem et al., 2013). Flaxseed oil contains polyunsaturated fatty acids, including omega‐3 and omega‐6, which are beneficial for health. It also contains lignans with health‐promoting properties and has been used to prevent acute alcoholic hepatic steatosis due to its anti‐inflammatory properties (Wang et al., 2016; Salem & Eggersdorfer, 2015).

Both olive oil and flaxseed oil contain active components like oleic acid, linoleic acid, and linolenic acid, which have antioxidant and anti‐inflammatory properties. They help reduce free radicals and oxidative stress, thereby lowering elevated liver enzyme levels. These oils are rich in omega‐3, omega‐6, and omega‐9 fatty acids, essential for overall health (Abenavoli et al., 2019).

The current study aimed to explore the Hepatoprotective potential of olive oil and flaxseed oil against hepatotoxicity. The cited studies provide scientific evidence supporting the use of these oils in reducing liver damage and elevated enzyme levels. Incorporating these oils into the diet can harness their antioxidant and anti‐inflammatory properties, promoting liver health and reducing the risk of hepatotoxicity.

## 2 MATERIALS AND METHODS

Olives and flaxseeds oil were purchased from local market of Faisalabad. All chemicals and reagents were availed at laboratory of the National Institute of Food Science and Technology, University of Agriculture, Faisalabad for the research and analyses purpose.

### 2.1 Analysis of sample

#### 2.1.1 Free fatty acids

The free fatty acid content was determined by following the method of AOAC (2019), with slight modifications, approximately 5 g of the oil or fat sample was weighted accurately using an analytical balance and transferred into a conical flask. Later on, 50 mL of ethyl alcohol (95%) was added to the flask containing the sample and ensure that the alcohol is neutralized by adding a few drops of phenolphthalein indicator and titrating with sodium hydroxide until a faint pink color persists for 30 s. The mixture was heated on a water bath while stirring until the sample completely dissolves in the alcohol. Overheating was avoided to prevent evaporation of alcohol. The solution was allowed to cool at room temperature, 1–2 drops of phenolphthalein indicator were added to the cooled solution. The solution was titrated with the standardized sodium hydroxide solution while continuously swirling the flask until a faint pink color that persists for at least 30 s appears. Calculations were done by given equation:

$$\text{Free fatty acids}as\text{oleic},\%=\frac{\text{m}\text{L}\text{of alkali}\times \text{M}\times 28.2}{\text{Mass, g of test portion}}$$

$$\text{Free fatty acids}as\text{palmitic},\%=\frac{\text{m}\text{L}\text{of alkali}\times \text{M}\times 25.6}{\text{Mass, g of test portion}}$$

#### 2.1.2 Iodine value

The iodine value was determined by following the method of AOAC (2019), briefly, fat was melted by gentle heating. Ensuring the temperature does not exceed 10°C to avoid decomposition, the melted sample was filtered through filter paper to remove any impurities, 0.2 g of melted sample was taken into Erlenmeyer flask. Later on, 10 mL of cyclohexane and 10 mL of glacial acetic acid (1:1 v/v) was added to the flask containing the sample, the flask was swirled to dissolve the sample completely, 25 mL of Wijs solution was added to the flask, the flask was placed in a dark place and allowed to stand for 30 min. After 30 min, 20 mL of 10% potassium iodide solution was added to the flask, following this 100 mL of distilled water was immediately added to the flask to stop the reaction. The mixture was titrated with standardized 0.1 N sodium thiosulfate solution. The sodium thiosulfate solution was added gradually while swirling the flask was continued until the yellow color of the iodine starts to fade. The solution becomes pale yellow, add a few drops of starch indicator solution, until the solution turned blue. Calculations were done by given equation:

$$\text{Iodine value}=\frac{\left(\text{B}–\text{S}\right)\times \text{M}\times 12.691}{\text{Mass, g of test portion, g}}$$

### 2.2 Experimental design and ethical approval

The hepatotoxicity was assessed on rat model. The research was carried out on rats for the duration of 28 days. For bio assessment, 30 healthy rats each weighing 150–250 g were housed in the animal room under normal conditions of light, humidity and temperature, rats were kept at Animal House of National Institute of Food Science and Technology, University of Agriculture Faisalabad. All rats were provided with standard conditions including ventilation facility, 12/12‐hour period of light dark cycle and temperature of room about (22 ± 2°C). Prior to the research experiment ethical approval was obtained from the Office of Research, Innovation and Commercialization. The ethical approval was obtained from the Directorate of Research and Innovation, University of Agriculture Faisalabad (Reference number 1445/ORIC dated 14‐05‐2021). Acclimatization period of 1 week prior to start the experimental study treatment with proper access to normal feed and water. The study was conducted by following the guidelines of animal care of National Biosafety Rules 2005, Punjab Biosafety Rules 2014, and Punjab Animal Act 2019 (Singh et al., 2020). Rats were divided in to five equal groups. Each group contained 6 rats. Group 1 consist of those rats who were fed normal diet and no disease occurring. Remaining other groups were hepatotoxic rats. Out of those four hepatotoxic groups one group was considered was control group. Which was basically disease group but they were fed on normal diet. Remaining three groups were hepatotoxic and they were given research substance. Group 3 was hepatotoxic group and it was given 4 mL/kg of flaxseed oil. Group 4 was hepatotoxic group and it was given 2.5 mL/kg olive oil. Last group was comparative group in which both oils given to hepatotoxic rats in 2 mL/kg flaxseed oil +1.25 mL/kg olive oil ratio. As showed in Table 1, the intervention groups according to treatment plan have been listed.

**TABLE 1:** Pattern of treatment plan.

Groups	Description
G_1_	No disease, Normal diet
G_2_	CCl_4_ induced hepatotoxicity group received with Normal diet
G_3_	CCl_4_ induced hepatotoxicity group received 4 mL/kg of flaxseed oil
G_4_	CCl_4_ induced hepatotoxicity group received 2.5 mL/kg olive oil
G_5_	CCl_4_ induced hepatotoxicity group received 2 mL/kg flaxseed oil +1.25 mL/kg olive oil for 4 weeks

### 2.3 Induction of hepatotoxicity

CCl_4_ (1 mL/kg) was used twice in first week to induce hepatotoxicity in rats by using method of Ekebas et al. (2019). For this purpose, after induction, CCL_4_ results were examined whether hepatotoxicity occurred or not. This process was done by observing elevated hepatic enzymes. Hepatic enzymes were examined by liver function test. It was observed that CCl_4_ should be given in permissible limit as high dosage in chemical induction can cause death of rat. The doses of 4 mL/kg for flaxseed oil and 2.5 mL/kg for olive oil were selected based on previous studies that demonstrated effective hepatoprotective properties at these concentrations (Sachan et al., 2021). Preliminary experiments conducted in our lab also indicated that these doses were well‐tolerated and produced significant biological effects in rats. The oils were administered orally using a gavage needle to ensure precise dosing. The oils were administered once daily starting one day after the induction of hepatotoxicity with CCl4 and continued for the duration of the study (28 days).

### 2.4 Feed efficiency ratio (FER)

Throughout the duration of experiment, feed intake was recorded daily, whereas body weight was measured on weekly basis. The net feed intake and body weight gain was calculated by using method of Solanki and Zaveri (2012).

### 2.5 Biochemical tests

#### 2.5.1 Measurement of serum lipid profile

The lipid profile; high density lipoprotein (HDL), total cholesterol (TC), low density lipoprotein (LDL) and triacylglycerol (TAG) were determined by the following the method of Senyigit et al. (2018).

#### 2.5.2 Liver function test

Enzymes ALT (alanine transaminase), Alkaline Phosphatase (ALP) and AST (Aspartate transaminase) were determined by the following the method of Senyigit et al. (2018).

### 2.6 Statistical analysis

The results of all parameters were stated as mean with standard deviation (Mean ± SD). By using one‐way analysis of variance (ANOVA) the data was statistically analyzed. Tuckey's test was applied among different treatment groups to make a comparison and to identify the statistical difference (Montgomery, 2017).

## 3 RESULTS AND DISCUSSION

### 3.1 Free fatty acid and iodine value of oil

Results showed for oleic acid in olive oil, the percentage of free fatty acids was found to be 0.89%. This low value indicates good quality and minimal hydrolytic rancidity, which is essential for consumer acceptance and the stability of the oil. For linoleic acid in flaxseed oil, the percentage of free fatty acids was 0.39%. This even lower value suggests excellent quality and stability, which is crucial given the higher susceptibility of flaxseed oil to oxidation due to its high polyunsaturated fatty acid content. However, the iodine value was 76.3 mg/100 g. This value is within the typical range for olive oil, indicating a moderate level of unsaturation, which contributes to its balanced stability and nutritional profile. For flaxseed oil, the iodine value was 173.9 mg/100 g. This high value reflects the high level of unsaturation, particularly the presence of alpha‐linolenic acid (omega‐3 fatty acid). While this makes flaxseed oil nutritionally valuable, it also means it is more prone to oxidation and requires careful storage.

### 3.2 Liver function test

#### 3.2.1 ALP

ALP is an important liver enzyme secreted by hepatocytes. Damage to hepatocytes can disrupt ALP levels, with elevated levels typically indicating chemical damage or liver injury. While minor damage is often recoverable, severe damage can lead to irreversible effects. The mean values of ALP levels obtained from the study are presented in Table 2. When carbon tetrachloride (CCl₄) was administered to the control group (G0), the ALP level significantly increased to 283.17 ± 1.16 U/L, compared to the normal value of 143.40 ± 4.38 U/L. This increase indicates substantial liver damage caused by CCl₄. In the experimental groups, where different treatments were applied, the ALP levels decreased. The G2 and G3 groups showed a reduction in ALP levels compared to the control group, indicating some hepatoprotective effects. However, the most notable improvement was observed in the G4 group, which received a blend of flaxseed and olive oil. In this group, the ALP level was reduced to 115.53 ± 0.44 U/L, which is even lower than the normal value, indicating significant hepatoprotection and recovery from liver damage. Selim et al. (2018) support the current research findings, attributing the effectiveness of both oils to their free radical scavenging properties. The blend of flaxseed and olive oil appears to be particularly effective in reducing liver enzyme levels, suggesting potent antioxidant and anti‐inflammatory effects that protect hepatocytes from damage.

**TABLE 2:** Effect of olive oil and flaxseed oil concentration of ALP, ALT, AST among rats.

Groups	Alkaline phosphatase (ALP) (U/L)	Alanine transferase (ALT) (U/L)	Aspartate aminotransferase (AST) (U/L)
G0	143.40 ± 4.38^B^	55.06 ± 2.73^B^	69.20 ± 3.55^B^
G1	283.17 ± 1.16^A^	164.67 ± 5.16^A^	260.33 ± 6.65^A^
G2	116.07 ± 0.94^C^	54.80 ± 8.93^B^	55.39 ± 0.51^CD^
G3	137.00 ± 2.10^B^	58.03 ± 0.20^B^	58.33 ± 0.41^C^
G4	115.53 ± 0.44^C^	50.77 ± 1.46^B^	52.12 ± 0.36^D^

*Note:* G_0_, No disease, Normal diet. G1, CCl_4_ induced hepatotoxicity group received with normal diet. G2, CCl_4_ induced hepatotoxicity group received 4 mL/kg of flaxseed oil. G3, CCl_4_ induced hepatotoxicity group received 2.5 mL/kg olive oil. G4, CCl_4_ induced hepatotoxicity group received 2 mL/kg flaxseed oil +1.25 mL/kg olive oil for 4 weeks. Significance level = *p* < 0.05, values were significantly different from each other as A, B, C and D.

#### 3.2.2 Alanine transferase (ALT)

ALT is a critical biomarker for liver fibrosis, and its levels rise significantly in response to liver cell damage. The mean ALT values for olive oil and flaxseed oil treatments in chemically induced hepatotoxic rats are presented in Table 2. In the group induced with CCl₄ to cause hepatotoxicity (G1), where no olive oil or flaxseed oil was administered, the ALT levels were significantly elevated to 164.67 ± 5.16 U/L. This is markedly higher compared to the negative control group, which had ALT levels of 55.06 ± 2.73 U/L, indicating substantial liver damage in the absence of any treatment. When flaxseed oil was administered to the hepatotoxic rats in group G2, the elevated ALT levels decreased to 54.80 ± 8.93 U/L, demonstrating a significant protective effect against liver damage. The most notable improvement was observed in group G4, which received a combination of both olive oil and flaxseed oil. In this group, ALT levels were reduced to 50.77 ± 1.46 U/L, indicating superior hepatoprotective effects and a remarkable reduction in liver enzyme levels. The findings of this study are supported by the research of Mohammadian et al. (2018) and Selim et al. (2018), who also reported the beneficial effects of olive oil and flaxseed oil in reducing liver damage markers. The combined use of these oils appears to enhance their hepatoprotective properties, likely due to their antioxidant and anti‐inflammatory activities.

#### 3.2.3 Aspartate aminotransferase (AST)

AST, also known as glutamic oxaloacetic transaminase, is another crucial enzyme whose levels rise in response to hepatocyte damage. Elevated AST levels can affect not only the liver but also other organs such as the brain and heart. The results of the current study, as presented in Table 2, highlight the effects of olive oil and flaxseed oil on AST levels in chemically induced hepatotoxic rats. In the positive control group (G1), which was induced with CCl₄ and received no treatment, the AST levels were significantly elevated. When flaxseed oil was administered to the hepatotoxic rats in group G2, the mean AST level decreased to 55.39 ± 0.51 U/L, demonstrating a substantial protective effect against liver damage. Similarly, the administration of olive oil in group G3 resulted in a reduction of AST levels to 58.33 ± 0.41 U/L. The most significant improvement was observed in group G4, which received a combination of both olive oil and flaxseed oil. In this group, AST levels were reduced to 52.12 ± 0.36 U/L, indicating the most effective hepatoprotective effect among the tested groups. The findings of this study are supported by the research of Selim et al. (2018) and Ekebas et al. (2019), who also reported the beneficial effects of olive oil and flaxseed oil in reducing liver enzyme levels and protecting against liver damage. The combined use of these oils appears to enhance their hepatoprotective properties, likely due to their antioxidant and anti‐inflammatory activities.

### 3.3 Lipid profile

#### 3.3.1 Cholesterol

The cholesterol concentration in blood is a crucial indicator of lipid metabolism and overall cardiovascular health. The results of the current study, as presented in Table 3, show the effects of olive oil and flaxseed oil on cholesterol levels in chemically induced hepatotoxic rats. In the chemical‐induced toxic group (G1), where CCl₄ was administered without any treatment, the cholesterol concentration in blood reached the highest value of 278.63 ± 2.01 mg/dL. This is significantly higher compared to the negative control group (G0), which had a cholesterol concentration of 160.57 ± 2.73 mg/dL. When flaxseed oil was administered to the hepatotoxic rats in group G2, the cholesterol concentration decreased to 148.14 ± 1.46 mg/dL, indicating a substantial reduction in cholesterol levels due to the flaxseed oil treatment. Similarly, the administration of olive oil in group G3 resulted in a reduction of cholesterol levels to 148.14 ± 1.46 mg/dL, demonstrating a comparable cholesterol‐lowering effect. The most significant improvement was observed in group G4, which received a combination of both olive oil and flaxseed oil. In this group, the cholesterol concentration was reduced to the lowest value of 135.4 ± 1.43 mg/dL, indicating the most effective reduction in cholesterol levels among the tested groups. The findings of this study are supported by the research of Ekebas et al. (2019), who also reported the cholesterol‐lowering effects of olive oil and flaxseed oil. The combined use of these oils appears to enhance their lipid‐lowering properties, likely due to their beneficial fatty acid profiles and antioxidant properties.

**TABLE 3:** Effect of olive oil and flaxseed oil concentration on Cholesterol, Triglycerides, LDL, HDL in rats.

Groups	Cholesterol (mg/dL)	Triglycerides (mg/dL)	Low density lipoprotein (LDL) (mg/dL)	High density lipoprotein (HDL) (mg/dL)
G0	160.57 ± 2.73 B	120.30 ± 1.23 C	81.31 ± 2.42 B	48.27 ± 0.32 B
G1	278.63 ± 2.01 A	250.49 ± 1.80 A	195.86 ± 1.46 A	29.89 ± 0.52 D
G2	148.14 ± 1.46 C	116.27 ± 1.77 D	58.55 ± 0.08 D	49.20 ± 0.45 B
G3	143.92 ± 0.75 D	123.35 ± 0.69 B	62.19 ± 0.67 C	54.04 ± 0.32 A
G4	135.4 ± 1.43 E	112.95 ± 1.57 E	57.14 ± 2.35 D	46.39 ± 1.67 C

*Note:* G_0_, No disease, Normal diet. G1, CCl_4_ induced hepatotoxicity group received with normal diet. G2, CCl_4_ induced hepatotoxicity group received 4 mL/kg of flaxseed oil. G3, CCl_4_ induced hepatotoxicity group received 2.5 mL/kg olive oil. G4, CCl_4_ induced hepatotoxicity group received 2 mL/kg flaxseed oil +1.25 mL/kg olive oil for 4 weeks. Significance level = *p* < 0.05, values were significantly different from each other as A, B, C and D.

#### 3.3.2 Triglycerides

The concentration of triglycerides in the blood is an important marker of lipid metabolism and cardiovascular health. The results of the current study, as shown in Table 6, highlight the effects of olive oil and flaxseed oil on triglyceride levels in chemically induced hepatotoxic rats. In the chemical‐induced toxic group (G1), where CCl₄ was administered without any treatment, the triglyceride concentration reached the highest value of 250.49 ± 1.80 mg/dL. This elevated level indicates significant disruption in lipid metabolism due to the hepatotoxic effects of CCl₄. The administration of flaxseed oil in group G2 resulted in a notable reduction in triglyceride levels, though the specific value is not mentioned here. Similarly, the group receiving olive oil (G3) also showed a reduction in triglyceride concentration, indicating the beneficial effects of these oils on lipid metabolism. The most significant improvement was observed in group G4, which received a combination of both olive oil and flaxseed oil. In this group, the triglyceride concentration was reduced to the lowest value of 112.95 ± 1.57 mg/dL, demonstrating the most effective reduction in triglyceride levels among the tested groups. The findings of this study are consistent with those of Sales et al. (2019) and Selim et al. (2018), who also reported similar triglyceride‐lowering effects of olive oil and flaxseed oil. The combined use of these oils appears to enhance their lipid‐lowering properties, likely due to their beneficial fatty acid profiles, antioxidant properties, and potential synergistic effects.

#### 3.3.3 LDL

The concentration of triglycerides in the blood is a crucial indicator of lipid metabolism and cardiovascular health. The results of the current study, presented in Table 3, demonstrate the effects of olive oil and flaxseed oil on triglyceride levels in chemically induced hepatotoxic rats. In the chemical‐induced toxic group (G0), where CCl₄ was administered without any treatment, the triglyceride concentration reached the highest value of 195.86 ± 1.46 mg/dL. This elevated level indicates significant disruption in lipid metabolism due to the hepatotoxic effects of CCl₄. In the negative control group (G1), which did not receive CCl₄ or any treatment, the triglyceride concentration was 81.31 ± 2.42 mg/dL, representing the baseline triglyceride level in healthy rats. When flaxseed oil was administered to the hepatotoxic rats in group G2, there was a notable reduction in triglyceride levels. Similarly, the administration of olive oil in group G3 resulted in a reduction in triglyceride concentration, indicating the beneficial effects of these oils on lipid metabolism. The most significant improvement was observed in group G4, which received a combination of both olive oil and flaxseed oil. In this group, the triglyceride concentration was reduced to the lowest value of 57.14 ± 2.35 mg/dL, demonstrating the most effective reduction in triglyceride levels among the tested groups. The findings of this study align with those reported by Sales et al. (2019) and Selim et al. (2018), who also observed significant triglyceride‐lowering effects of olive oil and flaxseed oil. The combined use of these oils appears to enhance their lipid‐lowering properties, likely due to their favorable fatty acid profiles, antioxidant properties, and potential synergistic effects.

#### 3.3.4 HDL

High‐density lipoprotein (HDL) is known as the “good” cholesterol due to its role in transporting fatty acids out of the liver and preventing arterial plaque buildup. The drug‐induced hepatotoxicity caused a significant reduction in HDL levels, disrupting normal lipid metabolism. However, the use of olive oil and flaxseed oil has shown a potential to restore HDL levels back to normal. The results presented in Table 3, demonstrate the effects of these oils on HDL levels in chemically induced hepatotoxic rats. The group (G0) that did not receive any drug showed the highest HDL level at 54.04 ± 0.32 mg/dL, indicating normal liver function and fat metabolism without any toxic interference. The group (G1) that received the hepatotoxic drug without any treatment had the lowest HDL level at 29.89 ± 0.52 mg/dL, highlighting the adverse effect of the drug on lipid metabolism. The rats (G2) treated with flaxseed oil showed a significant increase in HDL levels to 49.20 ± 0.45 mg/dL after 28 days, demonstrating the hepatoprotective effect of flaxseed oil in improving HDL levels. The study results indicate that both flaxseed oil and olive oil help mitigate the drug‐induced reduction in HDL levels, restoring them closer to normal levels. The most substantial increase in HDL levels was observed in the G2 group treated with flaxseed oil, indicating its effectiveness in promoting healthy lipid metabolism. These findings align with the studies by Ahmad et al. (2017) and Selim et al. (2018), which also reported similar HDL‐raising effects of olive oil and flaxseed oil. The beneficial impact of these oils is likely due to their high content of omega‐3 and omega‐6 fatty acids, antioxidants, and anti‐inflammatory properties.

### 3.4 Alteration in body weight

The current study monitored the body weight of rats on a weekly basis, as cirrhosis and liver injury are known to cause weight reduction in rats (Yan et al., 2023). The mean values of body weight alterations are presented in Table 4. Upon the induction of chemicals in the G1 group, a noticeable weight reduction was observed, with a mean value of 201.52 ± 1.84 g, starting from an initial weight of 202 g and reducing to 201 g after 28 days. The normal negative control group (G0) showed a mean weight reduction to 183.97 ± 3.46 g, indicating a minimal weight fluctuation without chemical induction. In contrast, the G2 group, which received flaxseed oil, exhibited a slight weight gain, reaching a mean weight of 198.53 ± 6.36 g from an initial weight of 189 g. Similarly, the G3 group, treated with olive oil, experienced an increase in body weight to 204.50 ± 0.3 g, surpassing their initial weight at the start of the trial. The G4 group, which received a combination of olive oil and flaxseed oil, also showed an increase in body weight, with a mean weight of 196.46 ± 5.40 g. These results suggest that the administration of olive oil and flaxseed oil, both individually and in combination, helped mitigate the weight loss typically associated with liver injury and cirrhosis. These findings are consistent with the studies by Selim et al. (2018) and Malik et al. (2017), which also reported protective effects of olive oil and flaxseed oil against weight reduction in hepatotoxic conditions.

**TABLE 4:** Effect of olive oil and flaxseed oil concentration on Body Weight on weekly bases (g) in rats.

Groups	7th	14th	21st	28th	Mean ± SD group
G_0_	179.50 ± 0.70^h^	182.83 ± 0.75^g^	185.17 ± 0.87^g^	188.37 ± 1.10^f^	183.97 ± 3.46 D
G_1_	202.21 ± 0.72^abc^	198.91 ± 0.46^d^	201.33 ± 0.42^bcd^	203.63 ± 0.32^ab^	201.52 ± 1.84 A
G_2_	189.03 ± 3.28^f^	201.65 ± 0.13^abcd^	198.95 ± 2.55^d^	204.50 ± 1.3^a^	198.53 ± 6.36 B
G_3_	191.0 ± 1.32^f^	198.95 ± 2.55^d^	199.67 ± 2.05^cd^	204.50 ± 0.3^a^	198.54 ± 5.26 B
G_4_	191.1 ± 0.52^f^	195.60 ± 4.57^e^	195.29 ± 2.93^e^	203.86 ± 1.57^ab^	196.46 ± 5.40 C
Mean Weeks	190.57 ± 7.60^c^	195.59 ± 7.18^b^	196.08 ± 6.24^b^	200.97 ± 6.59^a^	

*Note:* G_0_, No disease, Normal diet. G1, CCl_4_ induced hepatotoxicity group received with normal diet. G2, CCl_4_ induced hepatotoxicity group received 4 mL/kg of flaxseed oil. G3, CCl_4_ induced hepatotoxicity group received 2.5 mL/kg olive oil. G4, CCl_4_ induced hepatotoxicity group received 2 mL/kg flaxseed oil +1.25 mL/kg olive oil for 4 weeks. Significance level = *p* < 0.05, values were significantly different from each other as A, B, C and D.

### 3.5 Food intake measurement

The food consumption capacity of rats declined initially with the progression of chemically induced liver injury, but varied over time. The results of the mean variation in food intake are presented in Table 5. Throughout the experiment, weekly observations indicated changes in food intake across different groups. Group G0, the negative control group, showed consistent food intake. In contrast, Group G1, which received chemical CCl₄ for 28 days, exhibited a progressive increase in food intake over the 4 weeks: 14.70 ± 0.07 g/day in the 1st week, 15.87 ± 0.06 g/day in the 2nd week, 18.38 ± 0.15 g/day in the 3rd week, and 20.05 ± 0.02 g/day in the 4th week. This trend suggests that although food intake was initially low, it increased as the disease progressed. Group G2, subjected to chemical‐induced hepatotoxicity and treated with flaxseed oil, also demonstrated a gradual increase in food intake, as 14.61 ± 0.92 g/day in the 1st week, 15.75 ± 2.15 g/day in the 2nd week, 20.23 ± 1.03 g/day in the 3rd week, and 20.90 ± 0.22 g/day in the 4th week. Similarly, Group G3, which received chemical‐induced hepatotoxicity and was treated with olive oil, showed food intake values of 14.64 ± 0.16 g/day in the 1st week, 15.75 ± 2.15 g/day in the 2nd week, 20.23 ± 1.03 g/day in the 3rd week, and 20.03 ± 0.02 g/day in the 4th week. The most significant improvement was observed in Group G4, which was given a mixture of olive oil and flaxseed oil. This group had food intake values of 15.48 ± 0.72 g/day in the 1st week, 17.99 ± 1.49 g/day in the 2nd week, 19.48 ± 0.85 g/day in the 3rd week, and 23.24 ± 1.03 g/day in the 4th week. The food intake in G4 was consistently higher from the first week and continued to rise gradually, indicating that the combination of oils effectively enhanced the food consumption capacity in hepatotoxic rats. These findings are supported by the studies of Fadlalla et al. (2013) and Al‐Seeni et al. (2016), which reported similar beneficial effects of olive oil and flaxseed oil on food intake in hepatotoxic conditions.

**TABLE 5:** Effect of olive oil and flaxseed oil concentration on Food intake on weekly bases (g/day) in rats.

Groups	7th	14th	21st	28th	Mean ± SD Group
G_0_	14.75 ± 0.18^hi^	16.27 ± 0.12^fg^	19.33 ± 0.17^cd^	20.03 ± 0.02^bc^	17.59 ± 2.26 B
G_1_	14.70 ± 0.07^hi^	15.87 ± 0.06^gh^	18.38 ± 0.15^de^	20.05 ± 0.02^bc^	17.25 ± 2.18 BC
G_2_	14.61 ± 0.92^i^	15.75 ± 2.15^ghi^	20.23 ± 1.03^bc^	20.90 ± 0.22^b^	17.87 ± 3.05 B
G_3_	14.64 ± 0.16^hi^	15.65 ± 0.03^ghi^	17.23 ± 0.22^ef^	20.03 ± 0.02^bc^	16.89 ± 2.12 C
G_4_	15.48 ± 0.72^ghi^	17.99 ± 1.49^e^	19.48 ± 0.85^cd^	23.24 ± 1.03^a^	19.04 ± 3.07 A
Mean Weeks	14.83 ± 0.56^d^	16.31 ± 1.33^c^	18.93 ± 1.18^b^	20.85 ± 1.34^a^	

*Note:* G_0_, No disease, Normal diet. G1, CCl_4_ induced hepatotoxicity group received with normal diet. G2, CCl_4_ induced hepatotoxicity group received 4 mL/kg of flaxseed oil. G3, CCl_4_ induced hepatotoxicity group received 2.5 mL/kg olive oil. G4, CCl_4_ induced hepatotoxicity group received 2 mL/kg flaxseed oil +1.25 mL/kg olive oil for 4 weeks. Significance level = *p* < 0.05, values were significantly different from each other as A, B, C and D.

### 3.6 Fluid intake measurement

The fluid intake capacity of rats varied throughout the progression of chemically induced liver injury, with fluctuations observed on a weekly basis. This variation was influenced by factors such as weight reduction and changes in food intake capacity. The mean analysis presented in Table 6 illustrates these trends. In Group G0, the negative control group, fluid intake was highest in the first week at 32.96 ± 1.00 mL/day and decreased over time. In Group G1, which received CCl₄ to induce liver injury, fluid intake was 32.83 ± 0.78 mL/day in the 1st week, increased slightly to 32.98 ± 0.05 mL/day in the 2nd week, peaked at 34.32 ± 1.54 mL/day in the 3rd week, and then decreased to 31.75 ± 0.83 mL/day in the 4th week. This pattern indicates that fluid intake was initially high following the first dose of the drug but declined as the disease progressed. Group G2, which was given CCl₄ to induce hepatotoxicity and treated with flaxseed oil, showed fluid intake levels of 36.46 ± 1.49 mL/day in the 1st week, 33.03 ± 1.05 mL/day in the 2nd week, 27.22 ± 1.10 mL/day in the 3rd week, and 31.82 ± 1.21 mL/day in the 4th week. The administration of flaxseed oil appeared to mitigate some of the decline in fluid intake associated with liver injury. Similarly, Group G3, treated with olive oil, showed improved fluid intake: 36.53 ± 0.66 mL/day in the 1st week, 34.40 ± 0.45 mL/day in the 2nd week, 28.22 ± 0.90 mL/day in the 3rd week, and 31.15 ± 0.81 mL/day in the 4th week. Olive oil also helped in maintaining fluid intake levels despite the liver injury. Group G4, which received a combination of olive oil and flaxseed oil, demonstrated the most consistent fluid intake throughout the study: 35.84 ± 1.92 mL/day in the 1st week, 34.70 ± 0.79 mL/day in the 2nd week, 28.77 ± 0.63 mL/day in the 3rd week, and 32.82 ± 0.76 mL/day in the 4th week. This combination helped in maintaining fluid intake in liver‐injured rats more effectively than the individual oils. These findings are supported by similar trends observed in the studies of Al‐Seeni et al. (2016) and Yang et al. (2009), which also reported the beneficial effects of olive oil and flaxseed oil on fluid intake in hepatotoxic conditions.

**TABLE 6:** Effect of Olive oil and flaxseed oil concentration on Fluid intake on weekly bases (mL/day) in rats.

Groups	7th	14th	21st	28th	Mean ± SD Group
G_0_	32.96 ± 1.00^de^	32.1 ± 0.87^cde^	27.44 ± 0.71^g^	27.31 ± 0.60^g^	30.23 ± 3.06 C
G_1_	32.83 ± 0.78^de^	32.98 ± 0.05^de^	34.32 ± 1.54^bcd^	31.75 ± 0.83^ef^	32.97 ± 1.25 A
G_2_	36.46 ± 1.49^a^	33.03 ± 1.05^de^	27.22 ± 1.10^g^	31.82 ± 1.21^ef^	32.13 ± 3.61 B
G_3_	36.53 ± 0.66^a^	34.40 ± 0.45^bcd^	28.22 ± 0.90^g^	31.15 ± 0.81^f^	32.57 ± 3.35 AB
G_4_	35.84 ± 1.92^ab^	34.70 ± 0.79^bc^	28.77 ± 0.63^g^	32.82 ± 0.76^de^	33.03 ± 2.97 A
Mean Weeks	34.92 ± 2.03^a^	33.66 ± 0.98^b^	29.19 ± 2.85^d^	30.97 ± 2.10^c^	

*Note:* G_0_, No disease, Normal diet. G1, CCl_4_ induced hepatotoxicity group received with normal diet. G2, CCl_4_ induced hepatotoxicity group received 4 mL/kg of flaxseed oil. G3, CCl_4_ induced hepatotoxicity group received 2.5 mL/kg olive oil. G4, CCl_4_ induced hepatotoxicity group received 2 mL/kg flaxseed oil +1.25 mL/kg olive oil for 4 weeks. Significance level = *p* < 0.05, values were significantly different from each other as A, B, C and D.

## 4 CONCLUSION

The study conclusively demonstrates that olive oil and flaxseed oil possess significant hepatoprotective properties. Their administration, both individually and in combination, resulted in substantial improvements in liver enzyme levels and lipid profiles in CCl₄‐induced hepatotoxic rats. The combination of both oils provided the most pronounced benefits, suggesting a synergistic effect that enhances their protective properties. These findings support the potential use of olive oil and flaxseed oil as therapeutic agents for managing liver health, reducing lipid levels, and improving cardiovascular health.

## AUTHOR CONTRIBUTIONS

Waqas Ahmad Khan and Hina Rasheed designed the study and conduct under the supervision of Muhammad Inam‐ur‐Raheem and Muhammad Afzaal. Muhammad Abdullah Butt and Muhammad Arslan performed the study and participated in drafting the article with Noor Akram. Farhan Saeed helped in developing the whole concept and editing. Noor Akram and Gebremichael Gebremedhin Hailu helped in preparing Figures and Tables, the overall quality of the manuscript was maintained by Farhan Saeed. Aasma Asghar, Faiyaz Ahmed and Muhammad Afzaal wrote, edited and revised the manuscript critically. The final version of the manuscript has been read and approved by all listed authors.

## FUNDING INFORMATION

The authors declare that no funds, grants, or other support were received during the preparation of this manuscript.

## CONFLICT OF INTEREST STATEMENT

The authors declare that they have no conflict of interest.

## ETHICS STATEMENT

This article does not contain any studies with human participants or animals performed by any of the authors.

## CONSENT

For this type of study, formal consent is not required.

## Data Availability

Even though adequate data has been given in the form of tables and figures, however, all authors declare that if more data is required then the data will be provided on a request basis.
